# Convolutional Neural Networks for Estimation of Uniaxial Tensile Test Equivalent Properties from Small Punch Test

**DOI:** 10.3390/ma18235276

**Published:** 2025-11-22

**Authors:** Maciej Kaliciak, Tadeusz Uhl, Marek Nowak

**Affiliations:** 1 Faculty of Mechanical Engineering and Robotics, AGH University of Science and Technology, 30-059 Krakow, Poland; 2 Faculty of Space Technologies, AGH University of Science and Technology, 30-059 Krakow, Poland; tuhl@agh.edu.pl; 3 Office of Technical Inspection (UDT), 30-087 Krakow, Poland; marek.nowak@udt.gov.pl

**Keywords:** small punch test, uniaxial tensile test, neural network, machine learning, material properties, ultimate strength, yield strength

## Abstract

The Small Punch Test (SPT) has been developed as a small sample technique for the evaluation of mechanical properties of structural materials. However, the SPT is subject to systematic biases, resulting in inaccurate estimates of Uniaxial Tensile Test (UTT) properties. In this study, an experimental approach has been adopted to investigate the potential of neural networks to predict UTT-equivalent behavior from SPT measurements. An experimental database containing paired SPT and UTT data has been prepared for three boiler steels (10H2M, 13HMF, and 15HM) in both new and service-degraded states. Convolutional neural networks (CNN) have been trained and evaluated for curve-to-curve prediction. The working hypothesis is that CNN models, by exploiting local curve features, are capable of reducing the systematic bias inherent to SPT, generating estimates of UTT properties with precision comparable to conventional UTT measurements. Consistent with trends in applied deep learning, the results confirm the robustness of convolutional architectures. In general, the findings provide strong evidence that CNNs can translate SPT data into UTT equivalent material properties, thereby bridging a long-standing methodological gap and supporting automated evaluation of structural steels in service.

## 1. Introduction

The Small Punch Test (SPT) has been developed as a small sample technique for the evaluation of mechanical properties of structural materials. It has been investigated as an alternative or complementary method to classical approaches such as the Uniaxial Tensile Test (UTT) [1], with applications that extend to the estimation of the yield strength, ultimate tensile strength, creep properties, and ductile-to-brittle transition temperature (DBTT) [2,3,4]. This method has gained recognition because it requires only a minimal volume of material and can be applied to components in service, where the extraction of standard tensile samples is often not feasible. Due to these advantages, the SPT has attracted attention in power generation, aerospace, and civil engineering, where condition monitoring of critical components is essential to maintaining structural integrity and optimizing maintenance strategies.

In addition to its technical advantages, the SPT has become a valuable tool for the assessment of material degradation in steels exposed to service. Because degradation processes such as creep, fatigue, and thermal embrittlement manifest primarily via changes in mechanical properties, the SPT provides a practical way of identifying loss of strength or ductility without requiring large destructive samples. Numerous studies [2,5,6,7,8] have demonstrated its applicability for tracking degradation-related changes in yield strength, ultimate tensile strength, and DBTT, making the method an attractive candidate for integration into structural health monitoring frameworks.

Despite these benefits, direct correlation between SPT results and those obtained from conventional methods remains a challenge. The difficulty comes from the differences in stress states between the small punch configuration and the standard tensile loading. Although transformations have been proposed to convert parameters derived from SPT curves into their UTT equivalents, the resulting correlations have often been material-specific and not universally applicable [9,10,11]. Even in recent standards, normalized calculation procedures yield inconsistent accuracy across different steels, and repeated calibration remains necessary for industrial use. As a result, research has focused on complementary approaches that can reduce the need for empirical calibration.

Among such approaches, the use of machine learning, particularly neural network models, has gained attention. Early studies [12] demonstrated that artificial neural networks could approximate the inverse relationship between SPT load–displacement curves and material properties by training on data generated from finite element simulations, often based on the Gurson–Tvergaard–Needleman (GTN) damage model [13,14,15]. In these works, simulated SPT curves under varying input parameters served as training data, with neural networks used to reconstruct stress–strain behavior or estimate key mechanical properties. Studies using the Stuttgart Neural Network Simulator (SNNS) [16] and later backpropagation-based models confirmed the feasibility of this approach [17]. For boiler steels such as 10GN2MFA, 08Ch18N10T, and 14MoV6-3, reported average prediction errors were 1–3% for ultimate tensile strength and 4–8% for yield strength [17]. Although data generated from finite element (FE) simulations are invaluable for exploring mechanical responses under controlled conditions and for enriching limited experimental datasets [18,19], they may not always capture the stochastic variability present in real tests. In the present work, we intentionally relied solely on experimentally measured SPT and UTT data to ensure that the developed CNN model was validated on physical observed behavior rather than simulated approximations.

To address these shortcomings, recent research has begun to emphasize the use of experimental databases, although comprehensive studies remain scarce. The availability of paired SPT and UTT curves from the same material offers an opportunity to train neural networks directly on experimental evidence, bypassing the reliance on simulations. This shift is particularly important in the context of degradation monitoring, where service exposure introduces microstructural changes that are not easily replicated in simulations but are directly reflected in experimental data. At the same time, it provides a stringent test of the ability of neural networks to generalize under conditions of relatively limited dataset size, which remains a characteristic constraint in this domain.

Because the present work relies on a relatively small number of experimental data points, special attention was given to the prevention of overfitting and to the effective use of available data. In the broader machine learning literature, small-sample challenges are often addressed through techniques such as data augmentation, transfer learning, regularization, or architecture simplification. In this study, these limitations were mitigated by pairing all available SPT and UTT curves within the same material group to form unique training examples and applying dropout regularization throughout the CNN architecture. This strategy allowed the network to generalize effectively while preserving the physical consistency of the experimental database.

In the present study, an experimental approach has been adopted to investigate the potential of neural networks in order to predict the UTT-equivalent behavior from SPT measurements. An experimental database containing paired SPT and UTT data has been prepared for three boiler steels (10H2M, 13HMF, and 15HM) in both new and service-degraded states. Based on these data, a neural network architecture consisting of a convolutional neural network (CNN) designed for curve-to-curve prediction has been trained and evaluated. The working hypothesis is that by exploiting local curve features, CNN models are capable of reducing the systematic bias of SPT and providing more accurate property estimations.

The evaluation has been carried out with emphasis on two key aspects. First, the predicted force–displacement curves have been transformed into stress–strain data to allow for extraction of the yield strength and the ultimate tensile strength, with these values then compared directly to the UTT reference data. Second, validation procedures have been applied to assess the generalization of the model and identify the influence of outliers on the predictive accuracy. In line with the adopted criterion, predictions have been considered successful if they provide values closer to the UTT results than to the baseline SPT measurements.

By situating neural network predictions within this framework, the present work contributes to ongoing efforts to establish the SPT as a reliable technique for structural health assessment. Specifically, this paper positions the SPT not only as a miniature testing method but also as a viable route for automated evaluation of degradation through mechanical property assessment. In particular, it provides an experimental demonstration that CNN-based models can correct known biases of the SPT and deliver UTT-consistent predictions across multiple steels and degradation states. At the same time, the study highlights limitations related to yield strength detection and outlier sensitivity, indicating directions for future development. Through these contributions, this work aims to bridge the gap between the testing of miniature specimens and conventional mechanical characterization, offering a pathway toward the automated data-driven evaluation of structural steels in service.

Recent years have also seen a rapid expansion of machine learning (ML) and neural network (NN) applications in materials science beyond the Small Punch Test domain. Convolutional and fully connected architectures have been successfully employed to predict mechanical properties directly from hardness measurements or simplified mechanical inputs. Such data-driven approaches demonstrate the ability of NNs to capture complex nonlinear relations between material features and macroscopic strength parameters even in cases where traditional empirical models fail. The present study follows this trend by adapting a convolutional architecture for curve-to-curve translation between SPT and UTT responses, providing a new perspective on how ML can bridge miniature testing with standard tensile characterization.

During the past two decades, numerous attempts have been made to link SPT results with standard tensile parameters using models based on neural networks. Early approaches relied primarily on synthetic data obtained from finite element (FE) simulations. Abendroth [12] trained neural feed-forward networks on the output of the Gurson–Tvergaard–Needleman (GTN) model in order to approximate inverse mappings between the SPT curve and the material parameters for several steels. Linse [16] extended this concept using the Stuttgart Neural Network Simulator to reconstruct full SPT load–displacement curves through multiple displacement-specific networks. Subsequent studies [17,20] emphasized the need for large simulated datasets—often consisting of thousands of FE-generated curves—in order to achieve acceptable accuracy in predicting hardening or damage parameters. Despite these advances, such models remained limited by their reliance on idealized data and poor generalization across materials. More recent efforts have investigated various architectures, including multilayer perceptrons (MLP), convolutional neural networks (CNN), and Bayesian networks for different materials [18,19,21]. Although these studies have confirmed the potential of deep learning for mechanical property prediction, most of them used simulated input data and focused on scalar parameter estimation rather than full-curve reconstruction. In contrast, the present study employs a one-dimensional CNN trained exclusively on experimentally measured SPT and UTT data to achieve direct curve-to-curve translation. This approach extends previous machine learning applications beyond parameter identification, enabling physically interpretable prediction of complete UTT-equivalent responses from miniature SPT experiments.

In this paper, analyzed materials are introduced that are subjected to conventional and unconventional testing methods; in addition, a compositional approach is taken in order to correlate SPT and UTT. In the following chapters, this paper:

- Describes the evaluation of SPT capabilities to reflect degradation induced changes in mechanical properties.
- Describes the implementation, practical testing. results of a convolutional neural network for automation of SPT results interpretation.
- Further benchmarks CNN-based predictions against SPT results in the context of UTT correlation.

Based on the goals presented above, the following research questions have been answered:

- Can CNNs trained on paired SPT–UTT data outperform conventional SPT correlations in predicting UTT-equivalent curves?
- Can CNN-based models capture service-induced degradation effects across different steels?
- How robust are CNN predictions under experimental noise and outliers?

## 2. Materials and Methods

### 2.1. Materials

For this study, three boiler steels were used: 10H2M, 13HMF, and 15HM. The materials were chosen on the basis of market needs, adhering to the most commonly used steel types for pressure tanks and pipelines transporting pressurized steam. The selected materials were analyzed in two states: a new unused material group and a highly fatigued material group, with samples extracted from a material piece cut from construction that was excluded from further operation after reaching its contracted lifetime. The materials can be broken down into the following:

- 10H2M: D1—new; J1—250,000 h lifetime.
- 13HMF: M3—new; R1—200,000 h lifetime.
- 15HM: H1—new; T1—130,000 h lifetime.

Their mechanical properties [22,23,24,25] are shown in Table 1, while the compositions of the materials [22,23,24,25] are presented in Table 2.

Steel 10H2M (10CrMo9-10) [22] is a pearlitic alloy steel with high creep resistance, designed for use in elevated temperatures not exceeding 580 °C. A characteristic feature of this steel is the presence of M23C6 carbide and increased resistance to hydrogen in medium-pressure environments up to 500 °C. It is typically available in a heat-treated or normalized state.

Steel 15HM (13CrMo4-5) [22] is a low-alloy steel designed for use in high temperatures, distinguished by its low carbon content compared to other alloys with similar applications. It is characterized by good plasticity in both cold and hot working conditions as well as high machinability and weldability. It retains high mechanical strength at both room and elevated temperatures thanks to its resistance to aggressive environments, including temperatures up to 530 °C.

Steel 13HMF (14MoV6-3) [22] is a high-strength chromium-molybdenum steel designed for use at temperatures of 480–550 °C along with intense exposure to steam and industrial gasses. In its normalized state, it is used to manufacture seamless pipes for use in pressure and steam installations. An important alloying additive is vanadium, which improves strength properties and increases creep resistance compared to other boiler steels.

### 2.2. Experimental Methods

To obtain input data for neural network predictions and database building, a number of physical tests were conducted, resulting in a total yield of over 400 Small Punch Tests and 180 Uniaxial Tensile Tests.

#### 2.2.1. Small Punch Test

For this experiment, all SPT samples were tested according to the EN 10371:2021 standard [10], adhering to the test procedure with the exclusion of widening the allowed tolerances for the thickness of the sample from 0.5 mm ± 0.005 mm to 0.5 mm ± 0.01 mm, as supported by previous studies [26]. The sample diameter was kept at 8 mm, with standby tolerances and required specimen roughness. SPT tests were conducted on the TTS 190 machine [27] equipped with a load limit of 10 kN and a feed rate of 0.5 mm/s and a punch diameter of 1.25 mm. To transform raw data into comparable values, given equations are used to obtain key mechanical properties in the SPT. For assessment of the ultimate strength, Equation (1) is used, where: $R_{m}$ [MPa] is the ultimate strength; $\beta_{R_{m}}$ = 0.278 is the correlation factor; $F_{m}$ [N] is the break force; $h_{0}$ is the initial sample thickness [mm]; and $u_{m}$ is the sample deflection at the break point. For assessment of the yield strength, Equation (2) is used, where: $R_{e}$ [MPa] is the yield stress; $\beta_{R_{e}}$ = 0.51 is the correlation factor; $F_{e}$ [N] is the force at the point at the beginning of plastic deformation; and $h_{0}$ is the initial thickness of the sample [mm]. The two correlation factors are based on Annexes C and D of EN 10371, respectively [10].

(1) $$R_{m}=\beta_{R_{m}}\cdot \frac{F_{m}}{h_{0}\cdot u_{m}}$$

(2) $$R_{e}=\beta_{R_{e}}\cdot \frac{F_{e}}{h_{0}^{2}}$$

#### 2.2.2. Uniaxial Tensile Test

Similarly, our UTT experiments were conducted according to the ISO 6892-1 standard [1], with the total length of the specimen equal to 69 mm and an end rigged with M12 thread. The Z250 machine manufactured by Zwick Roell Group in Germany was used, with a maximum tensile force allowed of 250 kN and a preload value of 800 N.

### 2.3. Neural Network Application

**Figure 1:**
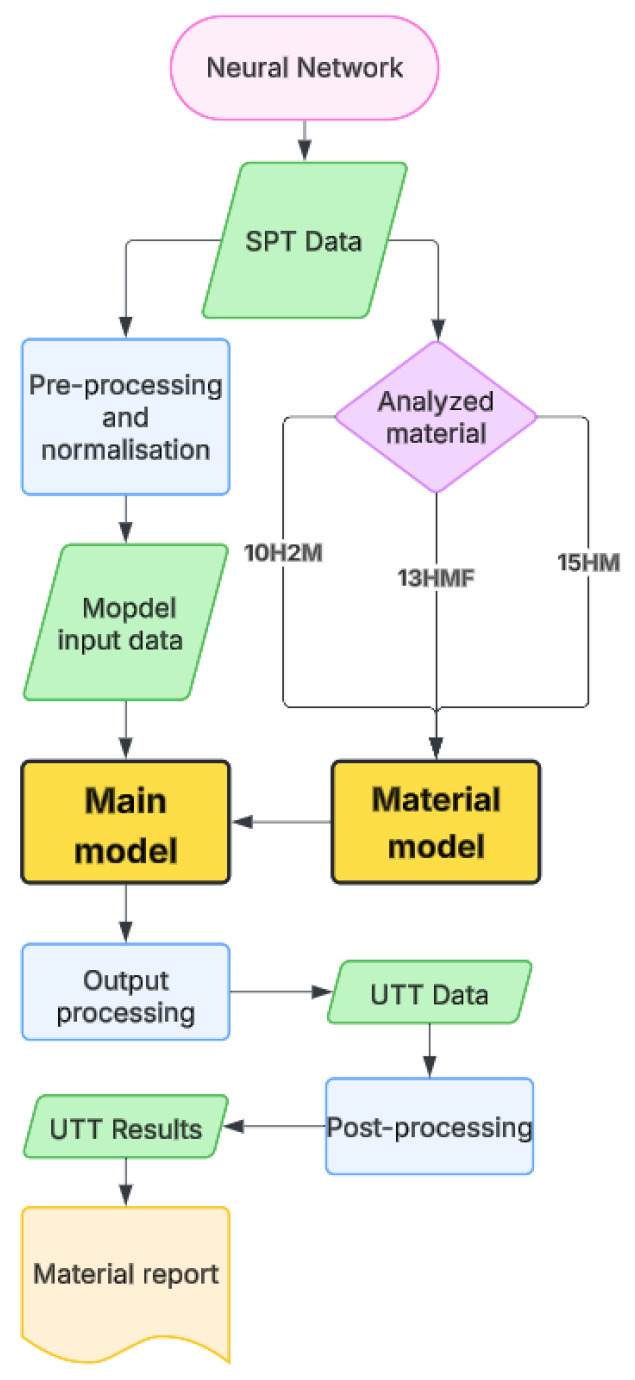
Neural network application block diagram.

A direct mapping from normalized Small Punch Test (SPT) force–displacement curves to normalized Uniaxial Tensile Test (UTT) force–displacement curves was learned. The supervised model ingested an entire SPT curve and predicted the entire UTT curve for the same material/state. Thus, finite element forward simulations were avoided, allowing for data-driven end-to-end reconstruction of UTT responses from suitable SPT measurements for operational use. All scripts were written in Python 3.12 programming language. The neural network framework (Figure 1) reproduce accepts raw SPT data provided in *.txt* format along with a material selection for analysis. It generates a stress–strain curve analogous to that obtained from UTT and extracts the corresponding mechanical parameters directly from the curve. The final results are intended for expert review, with manual verification recommended to ensure a reliable interpretation

#### 2.3.1. Database

##### Data Curation and Normalization

Raw laboratory exports were cleaned to remove metadata rows and columns, unify headers, standardize encoding, and harmonize column order across SPT and UTT. For modeling, both the SPT and the UTT curves were normalized to common scales determined from maxima throughout the repository (force and distance), which stabilized optimization and improved convergence. The normalized files retained the original units and identifiers in the metadata for full traceability. Prior to normalization and model training, all SPT and UTT curves were reviewed for data integrity. Curves exhibiting incomplete recordings, nonpositive force values, missing data points, or premature test termination were classified as noncompliant and excluded from further analysis. This ensured that only physically valid and fully recorded curves were used during neural network training and validation, maintaining the reliability of the resulting model.

##### Database Structure Determination

In this study, all SPT curves were paired with all available UTT curves within a given material group. This approach was motivated by the limited size of the experimental dataset, which was restricted to real test results. Unlike studies based on idealized or simulated curves [18,19], where large numbers of perfectly matched pairs can be generated, the present work was constrained by the high cost and effort of experimental testing. If the analysis were limited to strictly one-to-one pairings, the amount of usable data would have been insufficient to train a robust neural network.

Therefore, a many-to-many pairing design was adopted to expand the dataset and expose the model to a broader distribution of stress–strain responses observed within each material group. This allowed the network to learn more general features of the SPT–UTT relationship and account for the variability that is naturally present in the experimental results due to microstructural inhomogeneity, sample preparation, or alignment inaccuracies. In this way, the absence of simulated data was compensated for by allowing each curve to be interpreted in the context of all others belonging to the same steel grade, under the assumption that the mechanical behavior remains bounded within the distribution of properties typical for that material. This method prioritizes statistical robustness and model training stability over strict physical pairing. This choice reflects a deliberate balance made by the authors, dictated by the practical constraints of relying solely on experimental data without supplementation from simulated curves.

##### Curve-to-Curve Database Construction

A curve-to-curve database was prepared in SQLite [28]. Each record stored a full SPT curve paired with a full UTT curve from the same material state. Curves were serialized as JSON arrays consisting of normalized force and normalized distance, together with metadata on the material state labels, source filenames, and hashed curve IDs. Pairing was performed in a comprehensive manner within the material state categories to promote generalization, while the hashed IDs were used in split time to prevent leakage. The total number of samples imported into the database is shown in Table 3. To ensure reliable model training, SPT and UTT data from samples exhibiting testing errors, abnormal curve shapes, or clear procedural mistakes were excluded. The authors decided on this filtering process based on established experimental experience in order to prevent the neural network from learning spurious patterns arising from artifacts rather than from true material behavior.

##### Resampling

To enable efficient batching and consistent tensor shapes, each curve was resampled at points N=300 using an adaptive routine that increased the sampling density in regions of high variation. The first- and second-derivative magnitudes of the force distance signal were used to weight sample selection towards turning points and rapid transitions while versifying long and smooth segments. In this way, the critical shape information for the reconstruction of downstream stress–strain was preserved. The resulting C2C database retained the original set of pairs while reducing the storage from ∼3.8 GB to ∼166.6 MB.

##### Data Splits

The splits were performed at the *unique curve* level using hashed SPT and UTT IDs so that no curve appeared in more than one split, thereby eliminating pair-level leakage. A representative split used in the experiments is provided below. While this reduced the number of usable pairs in the training procedure, the absence of data leakage between sets ensured that the process remained robust and scientifically valid. Randomization was seeded and kept fixed in all runs to ensure reproducibility and fair comparison. The total of 3006 training pairs reported in Table 4 is the result of all possible pairings of SPT and UTT curves within the same material and degradation state, rather than being taken from additional experiments.

##### Material Awareness

Because SPT and UTT behavior depends on the material and degradation state, the material identity was encoded and concatenated to the input sequence. In the deployed variant, the network ingested two channels from the SPT curve (force, distance) and a material encoding broadcast along the sequence, yielding five input channels. This conditioning specialized the mapping without fragmenting the dataset by material.

#### 2.3.2. Neural Network Architecture

A one-dimensional convolutional neural network (1D CNN) was selected as the core architecture for curve-to-curve regression. This decision reflects the characteristics of the SPT and UTT data, which take the form of continuous smooth curves rather than sequences with long-range dependencies. Convolutional filters are well-suited to capturing local patterns such as the elastic regime, yield transition, and plastic hardening behavior, which are decisive for mechanical property estimation. Although convolutional neural networks are most commonly applied to two-dimensional image data, their one-dimensional variants have proven highly effective for sequential and signal-based tasks, including mechanical response modeling. Compared with recurrent neural networks or transformer-based architectures, the 1D CNN offers a favorable balance between accuracy, computational efficiency, and robustness in small-sample regimes thanks to its lower parameter count and stable convergence behavior. Dropout regularization and material-wise curve pairing were additionally employed to mitigate overfitting while preserving the physical interpretability of the extracted features.

##### CNN Model Description

A 1D convolutional neural network operating on length-300 sequences was used:

- **Backbone:** Six convolutional layers (kernel size k=7) with up to 512 hidden channels to capture multi-scale non-local dependencies in curve morphology.
- **Nonlinearity and regularization:** The risk of *overfitting* was particularly pronounced due to the limited size of the experimental database. To counteract this, *ReLU* activations were combined with a relatively high dropout rate (0.4) applied throughout the backbone, forcing the network to rely on more generalizable patterns rather than memorizing training data.
- **Input/Output tensors:** Input shape [C=5,L=300]; output shape [C=2,L=300], which produces the normalized UTT force–distance sequence.

The size of the kernel k=7 balanced locality with contextual reception along the curve, improving the fidelity of the turning point and the smoothness of the predicted sequence [29].

##### Training Objective and Optimization

The mean squared error (MSE) between the predicted and reference UTT sequences for both channels (averaged over time and batches) was minimized. AdamW with an initial learning rate of $10^{-3}$ was used for optimization [30,31]. A *ReduceLROnPlateau* scheduler reduced the learning rate when validation loss stagnated. Early stopping with best-checkpoint retention was applied to prevent overfitting and shorten runs without improvement. A batch size of 16 was adopted to stabilize the gradients under typical workstation constraints.

##### Hyperparameter Search and Fine-Tuning

**Figure 2:**
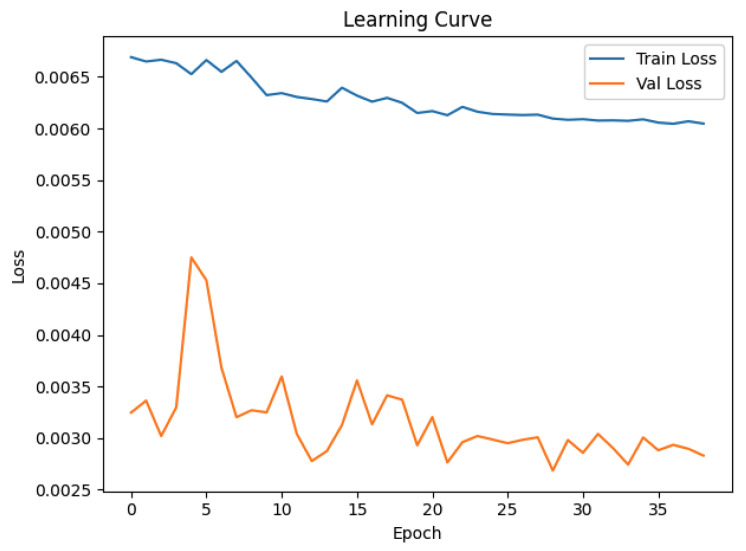
Neural network learning curve.

The optimization of the hyperparameters was performed with Optuna [32], starting with initial selection by a broad sweep (hidden layer size {64, 128, 256, 384, 512}, dropout [0.0;0.6]; batch size {8,16}, and activation {ReLU,LeakyReLU}), followed by a narrow search around the most promising region. Representative search runs used up to 50 epochs with a patience of 10. The best trial was then fine-tuned for up to 200 epochs at a reduced learning rate ($10^{-4}$) with weight decay $5\times 10^{-4}$ and scheduler patience 5, as shown in Figure 2; the best weights were overwritten in improvement to allow for safe interruption [29]. The entire table showing the exact values is available in Appendix A (Table A1).

To avoid overfitting and mitigate the effects of performance stagnation, the fine-tuned model was stored at the epoch corresponding to the minimum validation loss, preceding the onset of a prolonged plateau in the learning curve. This procedure ensured the preservation of the model state with optimal generalization.

#### 2.3.3. Inference and Postprocessing to Material Properties

The predicted normalized UTT sequences were denormalized to physical force–distance and converted to stress–stress using material-specific $S_{0}$ and $L_{0}$. The yield strength $R_{e}$ was calculated using the 0.2% offset method: an elastic slope was estimated from the initial linear segment, a line offset was constructed with 0.002 strain, and the intersection with the stress–strain curve was calculated. The ultimate tensile strength $R_{m}$ was taken as the maximum stress prior to cutting; this was because the generated curves ended at the start of the plastic, since the cutting was not modeled. Documented summaries and plots per sample were saved, and anomalous estimates were flagged for operator review.

#### 2.3.4. Evaluation Protocol

Performance was reported on the held-out test split using MAE, MSE, and $R^{2}$ calculated on the UTT sequences, and verified at the property level through $R_{e}$ and $R_{m}$ extracted from generated stress–strain curves. All test curves were strictly unseen, meaning that no hashed IDs overlapped with the training or validation sets. In addition to aggregate metrics, per-material breakdowns were produced to capture specialization effects introduced by material conditioning. Furthermore, confidence intervals for the mean were calculated using the Student’s *t*-distribution, which is appropriate when the population variance is unknown and the sample size is limited. The interval is defined as Equation (3):

(3) $$\bar{x}\;\pm \;t_{0.975,\;n-1}\;\frac{s}{\sqrt{n}},$$

where $\bar{x}$ is the sample mean, *s* is the sample standard deviation, and $t_{0.975,\;n-1}$ is the critical value of the *t*-distribution with n−1 degrees of freedom. In contrast, the classical *z*-interval assumes that the population standard deviation is known and that the sample size is large, assumptions that were not met in this study. Therefore, *t*-based confidence intervals were adopted to ensure reliable coverage of the true mean under the experimental conditions.

#### 2.3.5. Reproducibility

All code was implemented in Python version 3.12: PyTorch for modeling, Optuna for hyperparameter search, NumPy/pandas/SciPy for data and analysis, matplotlib for figures, and scikit-learn for metrics. Random seeds, immutable hashed-ID splits, pinned dependency versions, and checkpointed training runs were used to ensure repeatability. The SQLite/JSON C2C database, resampling routine, split lists, and training/evaluation scripts were all versioned and archived with the thesis materials to allow for independent reproduction [32,33,34,35,36,37,38]. All simulations and model training were performed on a standard personal computer Apple MacBook Air M1 model number A2337, manufactured in Zhengzhou, China in 2020 by Apple Inc. demonstrating that the proposed workflow can be reproduced on conventional engineering hardware without specialized computational resources. The final training of the neural network took approximately six hours. After training, the model performed inference and postprocessing operations without perceptible delay, enabling fully automated and near-realtime analysis of raw SPT input data.

#### 2.3.6. Outliers Removal

To ensure the robustness of the dataset, potential outliers were identified and removed using the modified Z-score method, which is based on the median absolute deviation (MAD). This approach is less sensitive to extreme values than conventional Z-scores, making it particularly suitable for experimental data with non-normal distributions. Data points with a modified Z-score that exceeded the commonly adopted threshold of ±3.5 were excluded from further analysis.

## 3. Results

### 3.1. Experimental SPT and UTT Results

The statistically evaluated experimental results are presented in the following paragraphs for each type of material used in this study. The results obtained for all three materials allowed us to successfully detect the ongoing degradation of the material, highlighting changes in mechanical properties that are more pronounced in the SPT results than in the conventional UTT method. This underlines the statement that SPT is more sensitive to detecting changes in yield strength in steel constructions subjected to corrosion, high pressures, and temperatures.

#### 3.1.1. Experimental Data of 10H2M Steel

For 10H2M steel (Table 5), SPT-derived ultimate tensile strength values closely matched UTT results, with only a minor upward shift of ∼15 MPa observed in the initial state. This consistency across material conditions indicates good reliability of the SPT method for assessing tensile strength. However, the yield strength showed a weaker correlation between the two methods. The SPT appeared to be more sensitive to microstructural changes, reflected in larger shifts and better detection of the plasticity threshold compared to the UTT.

#### 3.1.2. Experimental Data of 13HMF Steel

In the case of 13HMF steel (Table 6), the SPT results demonstrated very good agreement with the UTT results for both yield and ultimate tensile strength. Deviations between the methods remained within a few percent between both material states (new and degraded). In particular, for the tensile strength, the SPT provided values consistent with the UTT, confirming the robustness of the method. The statistical distributions indicated normality in most cases, suggesting stable repeatability of the measurement.

#### 3.1.3. Experimental Data of 15HM Steel

For 15HM steel (Table 7), SPT results reproduced the trends of UTT but with greater variability, especially in the yield strength of the degraded state, where several UTT outliers were identified. Although the tensile strength values obtained from the SPT were generally higher than those from the UTT, with up to ∼10% in the new state, the correspondence was still acceptable for comparative evaluation. The discrepancies in yield strength confirm the greater sensitivity of the SPT to local heterogeneity, but also highlight limitations in direct parameter comparability across methods.

### 3.2. CNN Validation and Prediction Performance

The curve-to-curve convolutional neural network was evaluated using a hold-out method for validation, with groups of samples split during training at the level of unique curve identifiers. The evaluation was conducted on strictly held-out curves, and the same automated postprocessing was applied to all predictions to obtain stress–strain curves and material properties ($R_{e}$, $R_{m}$). The following sequence-level metrics were calculated in the normalized domain and are presented in Table 8: mean absolute error (MAE), mean squared error (MSE), and coefficient of determination ($R^{2}$). Subsequent validation for each given material was conducted on the stress vs. strain curve level by evaluating predictions of the calculated mechanical properties.

#### 3.2.1. CNN Prediction of 10H2M Steel

For 10H2M steel (Table 9), the CNN model was validated on ten samples from each material group, namely, D1 and J1. The predictions reproduced the general population trend, with overlapping behaviors between the new and fatigued states. This reflects the subtle nature of the microstructural differences in 10H2M, which the CNN captured but did not always distinguish clearly.

The entire SPT population implemented into the CNN neural network resulted in predictions of the ultimate tensile strength that closely matched the UTT results, with the deviations remaining marginal. The yield strength showed larger discrepancies, particularly in the D1 group, where CNN predictions diverged from UTT values in the opposite direction compared to J1. These variations highlight CNN sensitivity to differences captured by SPT but not revealed by UTT, underlining the importance of analyzing the full predicted stress–strain curves rather than relying solely on extracted parameters (Table 10).

##### Stress–Strain Curve Without a Clear Yield Point

**Figure 3:**
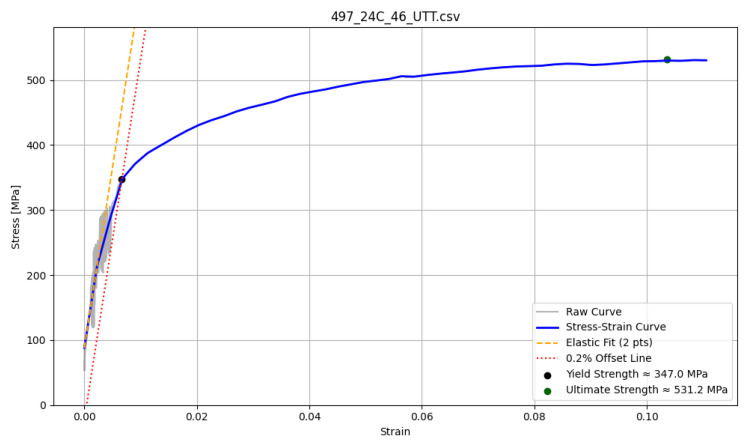
Example of 10H2M stress–strain curve without clear yield point.

An example of a stress–strain curve without a distinct yield point generated by CNN is shown in Figure 3. This illustrates the challenge faced by automated algorithms in determining the yield strength when a clear transition is absent. The implemented algorithm smooths the predicted curve to 50 points to reduce the noise introduced by the neural network. Subsequently, the standard 0.2% strain offset method is applied, with the elastic region taken as the baseline for the offset construction. A limitation of this approach is that the algorithm can interpret the curve geometry differently from an experienced researcher applying manual or geometrical methods to evaluate the yield strength.

#### 3.2.2. CNN Prediction of 13HMF Steel

For 13HMF steel (Table 11), the CNN model was evaluated on seven samples per group, M3 and R1. Overall deviations from UTT values remained within a few percent, although an outlier sample in the M3 group significantly affected prediction accuracy. This sample, already atypical in the experimental SPT test, led to an elevated predicted tensile strength and a reduction in the model’s R^2^ Excluding this outlier, the CNN showed very good predictive performance in both material states.

CNN predictions of the entire SPT population demonstrated excellent agreement with the UTT results, with yield strength deviations of approximately 1%. The clear yield behavior in UTT tests provided a strong training signal, enabling the CNN to effectively learn the characteristic saddle-shaped curve regions. The model achieved particularly high accuracy for the M3 group. However, seven R1 samples were classified as statistical outliers, reducing the effective size of the dataset and limiting overall performance in this group (Table 12).

#### 3.2.3. CNN Prediction of 15HM Steel

In the case of 15HM steel (Table 13), CNN validation included seven samples per group, H1 and T1. The model achieved high accuracy in reproducing strength values, with particularly precise predictions for the H1 group. As in the overall dataset, the CNN systematically underestimated the yield strength of the degraded material (T1) but maintained consistent and reliable predictions for the ultimate tensile strength.

The CNN model again produced highly accurate predictions of tensile strength for both both the H1 and T1 states based on the full population of the SPT. The yield strength predictions for the T1 group showed consistent underestimation relative to UTT, but with high repeatability and low variance. Closer inspection revealed that the UTT dataset itself contained five outliers in the T1 group, which biased the mean downwards. Compared to the full population average (406.5 MPa), the CNN predictions aligned more closely, suggesting that this apparent underestimate is largely attributable to anomalies in the experimental reference data rather than to model error (Table 14).

##### Stress–Strain Curve with Clear Yield Point

**Figure 4:**
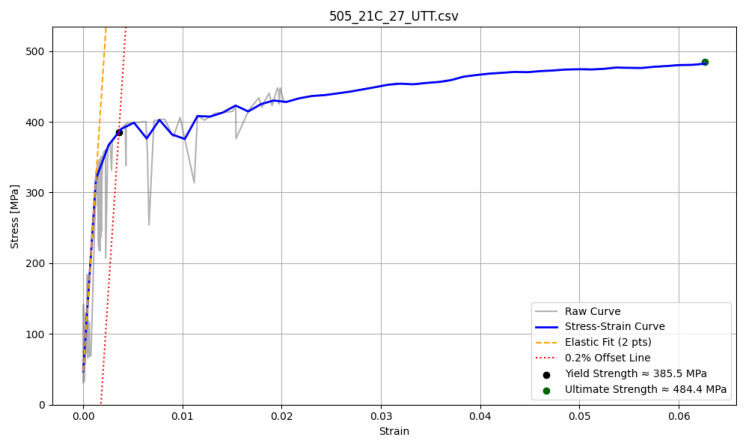
Example of 15HM stress–strain curve with clear yield point.

An example of a predicted and postprocessed stress–strain curve with a distinct yield point is presented in Figure 4. Although the standard 0.2% strain offset method is still applied for full automation, the visible transition greatly facilitates the final evaluation by the operator. In this case, the generated curve is less smooth than for materials without a clear yield point; nonetheless, the characteristic discontinuity remains evident, supporting reliable yield strength identification.

### 3.3. Confidence Intervals of NN Results

A summary of all the results of the validation test set is presented in Table 15, which highlights the confidence intervals in the results assessed on samples previously unseen by the neural network during training. The prediction uncertainty of the CNN model is represented by the 95% confidence intervals, calculated as described in Section 2.3.4.

### 3.4. Final Comparison: SPT and CNN Relative to UTT

The acceptance criterion for neural network predictions was defined such that the predicted material properties should align more closely with the UTT results than with the baseline SPT values. In all materials, the CNN model consistently met this requirement, showing the lowest average absolute deviation from UTT in yield strength predictions (2.9%), compared to 7.2% for SPT (Table 16). Although the estimation of the yield strength for 10H2M (D1) remained challenging due to the inherent difficulty of the algorithmic detection of the conventional yield point, the CNN predictions provided reproducible curves that support manual verification.

For ultimate tensile strength, the SPT systematically overestimated UTT values by 20–30 MPa, resulting in an average deviation of 4.9% (Table 17). In contrast, both neural networks achieved very good precision, with CNN predictions providing the closest correspondence between all material states.

## 4. Discussion

The results obtained in this study demonstrate that convolutional neural networks (CNNs) can serve as a reliable tool for predicting Uniaxial Tensile Test (UTT) properties from Small Punch Test (SPT) data. The working hypothesis was that SPT curves would provide estimates of the yield strength ($R_{e}$) and ultimate tensile strength ($R_{m}$) with precision comparable to conventional UTT measurements when processed through neural models. The present findings largely confirm this assumption, particularly for tensile strength, while also highlighting material- and state-dependent challenges for yield strength determination.

For tensile strength, CNN predictions consistently reproduced UTT reference values in all of the investigated steels (10H2M, 13HMF, and 15HM), with deviations limited to only a few percent. This precision exceeded that of the direct interpretation of SPT, which systematically overestimated the tensile strength by 20–30 MPa. Thus, the present study confirms that the SPT alone tends to bias absolute values, while data-driven models can correct for this offset. The high reproducibility of CNN output further supports their applicability in industrial contexts where precise estimation of tensile properties from miniature samples is a requirement.

In contrast, predicting the yield strength presented greater difficulty. The 10H2M steel proved difficult to assess due to the absence of a visible yield point inherited by CNN predictions, which demonstrated improved alignment with the UTT for other materials such as 13HMF and 15HM. This is shown on our example generated curves for both types of yield points. Our finding that the CNN systematically outperformed the SPT in its prediction of Re supports the hypothesis that convolutional architectures are better suited to capturing curve-level features, such as the saddle-shaped plateau that precedes yielding.

An important implication of these results is the sensitivity of neural networks to outliers. In 13HMF steel, an anomalous sample markedly affected the precision of the prediction and reduced the performance metrics $R^{2}$. This confirms that outlier management is critical when building training databases for small datasets. Although the decision to remove or retain outliers remains open, this work highlights the need for robust preprocessing protocols to prevent such data points from disproportionately influencing model generalization.

This study shows that convolutional neural networks (CNN) can reduce the systematic bias inherent in the SPT, generating estimates of material properties with a level of consistency that confirms their robustness for curve-to-curve prediction. The findings demonstrate that CNNs can effectively translate SPT data into UTT-equivalent stress–strain behavior, bridging a longstanding methodological gap and allowing for automated evaluation of boiler steels in service. This positions deep learning as a practical tool for material state assessment, particularly when direct tensile testing is impractical or destructive. At the same time, the results highlight persistent challenges, especially in yield strength prediction, which may benefit from hybrid physics-informed models and uncertainty quantification to increase reliability in safety-critical applications.

Nevertheless, the applicability of such networks remains largely material-specific. Models trained on 10H2M, 13HMF, and 15HM steels learn curve geometries and unique degradation patterns for these alloys, and cannot be assumed to generalize to other steels without adaptation. Factors such as differences in microstructure, deformation mechanisms, and sensitivity to boundary conditions limit the ability of the network to extrapolate beyond its training domain. To extend the proposed methodology, additional experimental datasets would be required for each new steel, allowing transfer learning or retraining of dedicated decoder heads. In this way, the approach could gradually evolve into a more universal framework for SPT-to-UTT translation; however, for now its use should be regarded as restricted to the same steels on which it was trained.

## 5. Conclusions

The present study has demonstrated that a one-dimensional convolutional neural network (1D CNN) can effectively translate the responses of the Small Punch Test (SPT) into the equivalent behavior of the Uniaxial Tensile Test (UTT). Our model captured characteristic curve features such as the yield transition and plastic hardening, providing accurate estimates of the yield strength ($R_{e}$) and the ultimate tensile strength ($R_{m}$) within experimentally validated confidence intervals. Compared with direct SPT interpretation, the CNN significantly reduced systematic bias and improved correlation with standard tensile results. The findings confirm the suitability of data-driven models for bridging miniature and conventional testing methods, supporting the development of automated tools for material degradation assessment. Future work will focus on expanding the experimental database, exploring transfer learning for other steel grades, and integrating physical constraints to improve the reliability of extrapolation.

## Figures and Tables

**Table 1:** Properties of the analyzed materials.

Material	Yield Strength [MPa]	Ultimate Strength [MPa]
10H2M	>300	480–630
13HMF	>300	460–610
15HM	>300	450–600

**Table 2:** Chemical material composition [%].

Material	C	Si	Mn	P	S	Cr	Mo	Ni	Cu	V	Al	N
10H2M	0.08–0.15	0.15-0.50	0.40–0.60	≤0.03	≤0.03	2.00–2.50	0.90–1.10	≤0.30	≤0.25	—	—	—
13HMF	0.10–0.18	0.15–0.35	0.40–0.70	≤0.04	≤0.04	0.3–0.6	0.5–0.65	≤0.3	≤0.25	0.22–0.35	≤0.02	—
15HM	0.11–0.18	≤0.35	0.40–0.70	≤0.025	≤0.01	0.70–1.15	0.40–0.55	≤0.35	≤0.25	—	—	≤0.012

**Table 3:** Total number of samples and pairs implemented in the *Curve-to-Curve* database.

Material	10H2M		13HMF		15HM	
Test	D1	J1	R1	M3	H1	T1
SPT	47	46	32	38	35	33
UTT	31	27	27	28	25	29
Sum of pairs	1457	1242	864	1064	875	957

**Table 4:** Data split of unique samples and data pairs for training, validation and testing of data in the CNN.

Dataset	Pairs	Unique SPT IDs	Unique UTT IDs
Train	3006	159	113
Val	72	24	18
Test	297	49	36

**Table 5:** SPT and UTT results summary for 10H2M steel.

Property	Material	Mean [MPa]	[%] AVG ∆ to UTT	Std Dev	AD Statistic	Shapiro *p*	Normality	Best-Fit Dist	N	Outliers Removed
$R_{e}$	D1 SPT	392	9.5%	34.2	0.9888	0.0202	Not Normal	gamma	68	0
$R_{e}$	D1 UTT	358	-	12	1.1796	0.0004	Not Normal	expon	31	0
$R_{e}$	J1 SPT	311	−5.8%	26.2	0.8289	0.8326	Normal	norm	67	2
$R_{e}$	J1 UTT	330	-	9.6	0.4429	0.6313	Normal	norm	29	1
$R_{m}$	D1 SPT	541	2.9%	14.7	0.7093	0.0925	Normal	norm	60	0
$R_{m}$	D1 UTT	526	-	11.6	0.8759	0.0289	Not Normal	weibull_min	31	0
$R_{m}$	J1 SPT	542	3.0%	15.7	1.4304	0.0004	Not Normal	weibull_min	65	4
$R_{m}$	J1 UTT	526	-	4.5	0.4755	0.3688	Normal	norm	30	0

**Table 6:** SPT and UTT results summary for 13HMF steel.

Property	Material	Mean [MPa]	[%] AVG ∆ to UTT	Std Dev	AD Statistic	Shapiro *p*	Normality	Best-Fit Dist	N	Outliers Removed
$R_{e}$	M3 SPT	357	−7.0%	16.7	0.5694	0.1901	Normal	norm	44	2
$R_{e}$	M3 UTT	384	-	7.1	0.4931	0.2487	Normal	norm	28	2
$R_{e}$	R1 SPT	437	−2.5%	28.3	0.7690	0.0479	Not Normal	weibull_mi	32	0
$R_{e}$	R1 UTT	448	-	9.7	0.0848	0.9996	Normal	norm	28	1
$R_{m}$	M3 SPT	558	6.3%	12.6	0.5941	0.1428	Normal	norm	44	2
$R_{m}$	M3 UTT	525	-	6.4	0.2379	0.9163	Normal	norm	28	2
$R_{m}$	R1 SPT	650	1.9%	19.4	0.4982	0.0885	Normal	norm	32	0
$R_{m}$	R1 UTT	638	-	10.9	0.3323	0.7139	Normal	norm	29	0

**Table 7:** SPT and UTT results summary for 15HM steel.

Property	Material	Mean [MPa]	[%] AVG ∆ to UTT	Std Dev	AD Statistic	Shapiro *p*	Normality	Best-Fit Dist	N	Outliers Removed
$R_{e}$	H1 SPT	306	−16.6%	15	0.4509	0.5472	Normal	norm	33	2
$R_{e}$	H1 UTT	367	-	13.5	1.0143	0.0026	Not Normal	weibull_mi	28	2
$R_{e}$	T1 SPT	410	−1.9%	46.4	0.3044	0.5684	Normal	norm	33	0
$R_{e}$	T1 UTT	418	-	21.2	1.6097	0.001	Not Normal	lognorm	25	5
$R_{m}$	H1 SPT	517	5.7%	8.8	0.8299	0.012	Not Normal	beta	31	2
$R_{m}$	H1 UTT	489	-	10	0.5645	0.1127	Normal	norm	28	2
$R_{m}$	T1 SPT	593	9.6%	18.5	0.3326	0.5556	Normal	norm	31	0
$R_{m}$	T1 UTT	541	-	15	0.8116	0.0508	Normal	norm	30	0

**Table 8:** CNN sequence-level validation performance.

Material	MAE	MSE	R^2^
D1	0.0513	0.0038	0.9422
H1	0.0224	0.0010	0.9535
J1	0.0437	0.0028	0.9692
M3	0.0866	0.0155	0.7803
R1	0.0186	0.0006	0.9543
T1	0.0186	0.0007	0.9516
Average	0.0402	0.0041	0.9252

**Table 9:** CNN validation mean results derived from predicted material properties of 10H2M steel compared with UTT.

Material	D1				J1			
**Property**	$R_{e}$		$R_{m}$		$R_{e}$		$R_{m}$	
**Unit**	**[MPa]**	**∆ [%]**	**[MPa]**	**∆ [%]**	**[MPa]**	**∆ [%]**	**[MPa]**	**∆ [%]**
UTT	358	-	526	-	330	-	526	-
CNN	336	−6.1%	529	0.5%	319	−3.4%	518	−1.6%

**Table 10:** Prediction results comparison table for 10H2M steel.

Property	Material	Mean [MPa]	Median [MPa]	Std Dev	[%] AVG ∆ to UTT	N	Outliers Removed	Usable N [%]
$R_{e}$	D1 CNN	330	328	17.23	−7.8%	47	0	100%
$R_{e}$	D1 UTT	358	348	32.02	-	31	0	100%
$R_{e}$	J1 CNN	337	341	16.65	2.1%	43	3	93%
$R_{e}$	J1 UTT	330	331	9.57	-	29	1	97%
$R_{m}$	D1 CNN	529	529	4.45	0.6%	47	0	100%
$R_{m}$	D1 UTT	526	523	11.57	-	31	0	100%
$R_{m}$	J1 CNN	523	522	3.41	−0.6%	41	5	89%
$R_{m}$	J1 UTT	526	525	4.52	-	30	0	100%

**Table 11:** CNN validation mean results derived from predicted material properties of 13HMF steel compared with UTT.

Material	M3				R1			
**Property**	$R_{e}$		$R_{m}$		$R_{e}$		$R_{m}$	
**Unit**	**[MPa]**	**∆ [%]**	**[MPa]**	**∆ [%]**	**[MPa]**	**∆ [%]**	**[MPa]**	**∆ [%]**
UTT	384	-	525	-	448	-	638	-
CNN	390	1.7%	545	3.7%	439	−1.9%	645	1.1%

**Table 12:** Prediction results comparison table for 13HMF steel.

Property	Material	Mean [MPa]	Median [MPa]	Std Dev	[%] AVG ∆ to UTT	N	Outliers Removed	Usable N [%]
$R_{e}$	M3 CNN	386	386	3.75	0.5%	36	2	95%
$R_{e}$	M3 UTT	384	384	7.09	-	28	2	93%
$R_{e}$	R1 CNN	458	458	4.14	2.2%	25	7	78%
$R_{e}$	R1 UTT	448	448	9.66	-	28	1	97%
$R_{m}$	M3 CNN	530	530	2.66	1.0%	36	2	95%
$R_{m}$	M3 UTT	525	525	6.41	-	28	2	93%
$R_{m}$	R1 CNN	642	641	4.92	0.6%	31	1	97%
$R_{m}$	R1 UTT	638	636	10.94	-	29	0	100%

**Table 13:** CNN validation mean results derived from predicted material properties of 15HM steel compared with UTT.

Material	H1				T1			
**Property**	$R_{e}$		$R_{m}$		$R_{e}$		$R_{m}$	
**Unit**	**[MPa]**	**∆ [%]**	**[MPa]**	**∆ [%]**	**[MPa]**	**∆ [%]**	**[MPa]**	**∆ [%]**
UTT	367	-	489	-	418	-	541	-
CNN	365	−0.5%	479	−2.0%	399	−4.5%	547	1.1%

**Table 14:** Prediction results comparison table for 15HM steel.

Property	Material	Mean [MPa]	Median [MPa]	Std Dev	[%] AVG ∆ to UTT	N	Outliers Removed	Usable N [%]
$R_{e}$	H1 CNN	364	364	2.12	−0.8%	33	2	94%
$R_{e}$	H1 UTT	367	369	13.46	-	28	2	93%
$R_{e}$	T1 CNN	401	400	5.95	−4.1%	32	1	97%
$R_{e}$	T1 UTT	418	425	21.24	-	25	5	83%
$R_{m}$	H1 CNN	482	481	1.6	−1.4%	33	2	94%
$R_{m}$	H1 UTT	489	490	10.05	-	28	2	93%
$R_{m}$	T1 CNN	548	547	3.29	1.3%	31	2	94%
$R_{m}$	T1 UTT	541	545	14.95	-	30	0	100%

**Table 15:** Mean values of $R_{e}$ and $R_{m}$ with 95% confidence intervals for each material group validation test set.

Material	$R_{e}$ [MPa]	$R_{m}$ [MPa]
D1	336.22 [321.46, 350.98]	528.56 [522.49, 534.63]
H1	365.23 [358.04, 372.42]	479.27 [476.60, 481.95]
J1	318.68 [298.39, 338.96]	517.74 [513.99, 521.49]
M3	390.44 [378.71, 402.18]	544.59 [505.43, 583.76]
R1	439.37 [401.35, 477.39]	645.25 [634.70, 655.79]
T1	399.28 [394.89, 403.67]	546.86 [538.22, 555.51]

**Table 16:** Mean Re Deviation of SPT and CNN in Comparison to UTT Results [%].

Group	J1	D1	M3	R1	H1	T1	ABS AVG
SPT	−5.8	9.5	−7.0	−2.5	−16.6	−1.9	**7.2**
CNN	2.1	−7.8	0.5	2.2	−0.8	−4.1	**2.9**

**Table 17:** Mean Rm Deviation of SPT and CNN in Comparison to UTT Results [%].

Group	J1	D1	M3	R1	H1	T1	ABS AVG
SPT	3.0	2.9	6.3	1.9	5.7	9.6	**4.9**
CNN	−0.6	0.6	1.0	0.6	−1.4	1.3	**0.9**

## Data Availability

Data supporting the reported results can be found in the AGH University of Kraków RODBUK repository under DOI: 10.58032/AGH/MCYRAQ [39].

## Abbreviations

The following abbreviations are used in this manuscript:

SPT	Small Punch Test
UTT	Uniaxial Tensile Test
CNN	Convolutional Neural Network
NN	Neural Network
C2C	Curve-to-Curve

## Appendix A

Appendix A summarizes the final configuration and tuning protocol of the one-dimensional convolutional neural network (1D CNN) used for curve-to-curve prediction. The purpose of this appendix is to provide full methodological transparency and reproducibility while maintaining conciseness in the main text. The network parameters listed in Table A1 correspond to the best-performing configuration obtained after hyperparameter search. The tuning process included systematic variation of the hidden layer width, dropout rate, learning rate, weight decay, and batch size followed by fine-tuning using the *ReduceLROnPlateau* scheduler and an early-stopping criterion. All runs were performed with fixed random seeds to ensure determinism and split integrity; the complete optimization logs together with the source code are available in the accompanying public database referenced in the manuscript [39].

**Table A1:** Final CNN hyperparameters and training configuration.

Component	Final Setting
Input length	L=300 (adaptive resampling)
Input channels	$C_{\mathrm{in}}=5$ (SPT force, SPT distance, material encoding)
Output channels	$C_{\mathrm{out}}=2$ (UTT force, UTT distance)
Conv layers	6 (1D)
Kernel size	7
Hidden channels	512
Activation	ReLU
Dropout	0.4 (throughout backbone)
Loss	Mean Squared Error (sequence-wise)
Optimizer	AdamW
Initial learning rate	$1\times 10^{-3}$
Scheduler	ReduceLROnPlateau
Fine-tune learning rate	$1\times 10^{-4}$
Weight decay (fine-tune)	$5\times 10^{-4}$
Batch size	16
Epochs (search/fine-tune)	up to 50/up to 200
Early stopping	Enabled; best-checkpoint retention
Split integrity	Unique hashed SPT/UTT IDs per split
Random seed	42—Fixed for all runs
